# Competing ferro- and antiferromagnetic exchange drives shape-selective $\hbox{Co}_3\hbox{O}_4$ nanomagnetism

**DOI:** 10.1038/s41598-020-77650-6

**Published:** 2020-12-02

**Authors:** Michael Shepit, Vinod K. Paidi, Charles A. Roberts, Johan van Lierop

**Affiliations:** 1 Department of Physics and Astronomy, University of Manitoba, Winnipeg, MB R3T 2N2 Canada; 2 Toyota Motor Engineering and Manufacturing North America Inc., 1555 Woodridge Avenue, Ann Arbor, MI 48105 USA; 3 Manitoba Institute for Materials, University of Manitoba, Winnipeg, MB R3T 2N2 Canada

**Keywords:** Magnetic properties and materials, Ferromagnetism, Nanoparticles

## Abstract

We have synthesized three different shapes of $\hbox{Co}_{3}\hbox{O}_{4}$ nanoparticles to investigate the relationships between the surface Co$^{2+}$ and Co$^{3+}$ bonding quantified by exploiting the known exposed surface planes, terminations, and coordiations of $\hbox{Co}_{3}\hbox{O}_{4}$ nanoparticle spheres, cubes and plates. Subsequently this information is related to the unusual behaviour observed in the magnetism. The competition of exchange interactions at the surface provides the mechanism for different behaviours in the shapes. The cubes display weakened antiferromagnetic interactions in the form of a spin-flop that occurs at the surface, while the plates show distinct ferromagnetic behaviour due to the strong competition between the interactions. We elucidate the spin properties which are highly sensitive to bonding and crystal field environments. This work provides a new window into the mechanisms behind surface magnetism.

## Introduction

The nanomagnetism in spinel oxides is wonderfully sensitive to the surface ions bond nature, thus providing a probe to the interactions at the surface. Ideally, one wishes to examine the surface spin magnetism by removing the magnetic contributions from the core. Via structural and magnetic characterizations from bulk and of the different surface terminations, the surface magnetism can provide a quantitative measure of the interionic exchange, and can then be mapped onto the surface chemistry e.g. bonding and coordination environments. This allows one to track exchange interactions and magnetism, such as ferromagnetism and the competition between ferro- and antiferromagnetic exchange interactions at the surface. In brief, $\hbox{Co}_{3}\hbox{O}_{4}$ has a normal spinel structure with magnetic Co$^{2+}$ ions located at tetrahedral sites and non-magnetic Co$^{3+}$ ions at octahedral sites. Even though Co$^{3+}$ is non-magnetic, it is essential to the overall magnetism by virtue of its presence in the extended superexchange pathway of ${\text{ Co}}^{{2+}} {{-}}{\text{O}}{{-}}{\text{Co}}^{{{\text{3+}}}} {{-}}{\text{O}}{{-}}{\text{Co}}^{{{\text{2+}}}}$, with Co$^{3+}$ ions located at the intermediary sites between O$^{2-}$ ions. Co$^{3+}$ ions are known to be key players in the magnetic properties, so the intrinsic behaviour should be highly susceptible to changes in the Co$^{2+}$:Co$^{3+}$ ratio^1,2^.

Quantifying the exchange interactions present in bulk and at different surface terminations can give us new insights into the ions’ behaviours and coordinations present. This is particularly true for $\hbox{Co}_{3}\hbox{O}_{4}$ where the exchange is a result of both Co$^{2+}$ and Co$^{3+}$ ions located at the different sites. In $\hbox{Co}_{3}\hbox{O}_{4}$ there are four superexchange pathways: The typical superexchange interaction through $\text{ Co}^{2+} {{-}}\text{ O } {{-}}\text{ Co}^{2+}$ ions, and three other exchange paths that involve Co$^{2+}$–O–Co$^{3+}$–O–Co$^{2+}$. The Co$^{2+}$–O–Co$^{2+}$ exchange is weak due to the larger distances associated with some of the O$^{2-}$ and Co$^{2+}$ ions, as first identified by Roth^2^. On the other hand, individual Co$^{2+}$–O–Co$^{3+}$–O–Co$^{2+}$ paths also have weak exchange, the multiplicity of the different paths between Co$^{2+}$ ions leads to an overall exchange strength that is not negligible—we define the multiplicity to be the total number of possible interaction paths from a Co$^{2+}$ ion to all neighbours of a given type (e.g. nearest or next nearest neighbours). This description follows that by Roth^3^ using a range of spinel oxides. For example, the bulk structural analogue of $\hbox{Co}_{3}\hbox{O}_{4}$, $\hbox{CoAl}_{2}\hbox{O}_{4}$, has a much smaller exchange ($J_{ij}/k_{B} = 0.4~\text{ K }$) compared to bulk $\hbox{Co}_{3}\hbox{O}_{4}$ ($J_{ij}/k_{B} = 4~\text{ K }$). However, $\hbox{CoAl}_{2}\hbox{O}_{4}$ contains Co$^{2+}$ ions located at tetrahedral sites and Al$^{3+}$ ions located at octahedral sites (instead of Co$^{3+}$ in $\hbox{Co}_{3}\hbox{O}_{4}$). The intervening Al$^{3+}$ ion *p*-orbitals get repelled to a higher energy state by the *p*-orbitals of the O$^{2-}$ ions, and the exchange pathway through the Al$^{3+}$ becomes unavailable. By comparison to the $\hbox{CoAl}_{2}\hbox{O}_{4}$ analogue, the Co$^{2+}$–O–Co$^{3+}$–O–Co$^{2+}$ paths account for $\sim$90% of the total exchange in $\hbox{Co}_{3}\hbox{O}_{4}$. By examining the different surface terminations—facilitated by the exchange interactions that propagate through the non-magnetic Co$^{3+}$ ion, characterization of the nanomagnetism results in direct quantification of the different site environments that are correlated to surface Co$^{2+}$:Co$^{3+}$ ratios.

The balance of Co$^{2+}$ and Co$^{3+}$ ions on the surface and their coordinations in these extended exchange pathways becomes very important as a crystal’s size decreases into the nanoscale regime. Here, we would expect measurable contributions from the surface atoms having a marked effect on the overall magnetic properties^4,5^. For example, at the nanoscale (e.g. in thin film and nanocrystallite form) broken exchange pathways at surfaces and interfaces result in significant changes in the Co$^{2+}$:Co$^{3+}$ ratios, and the local coordination around these ions affecting crystal field effects. This results in a decrease in the strength of the overall antiferromagnetic exchange, competition between the intrinsic antiferromagnetic and ferromagnetic exchange paths (as they can be quite different from the bulk) can induce a wide range of magnetic properties, from an antiferromagnetic spin-flop to ferromagnetic-like behaviour^6–8^.

With the ability to control the shape of $\hbox{Co}_{3}\hbox{O}_{4}$ nanoparticles, one can examine the impact on the magnetic properties from the exposed surfaces that present different Co$^{2+}$/Co$^{3+}$ ratios via the resultant surface terminations. That is, shape-selective control over the intrinsic properties. Specifically, by changing the surface terminations, we expect to reveal how the different magnetic properties are a result of the re-tuned environments, and how these environments are realized through the magnetism.

## Results

### Shape and composition

**Figure 1:**
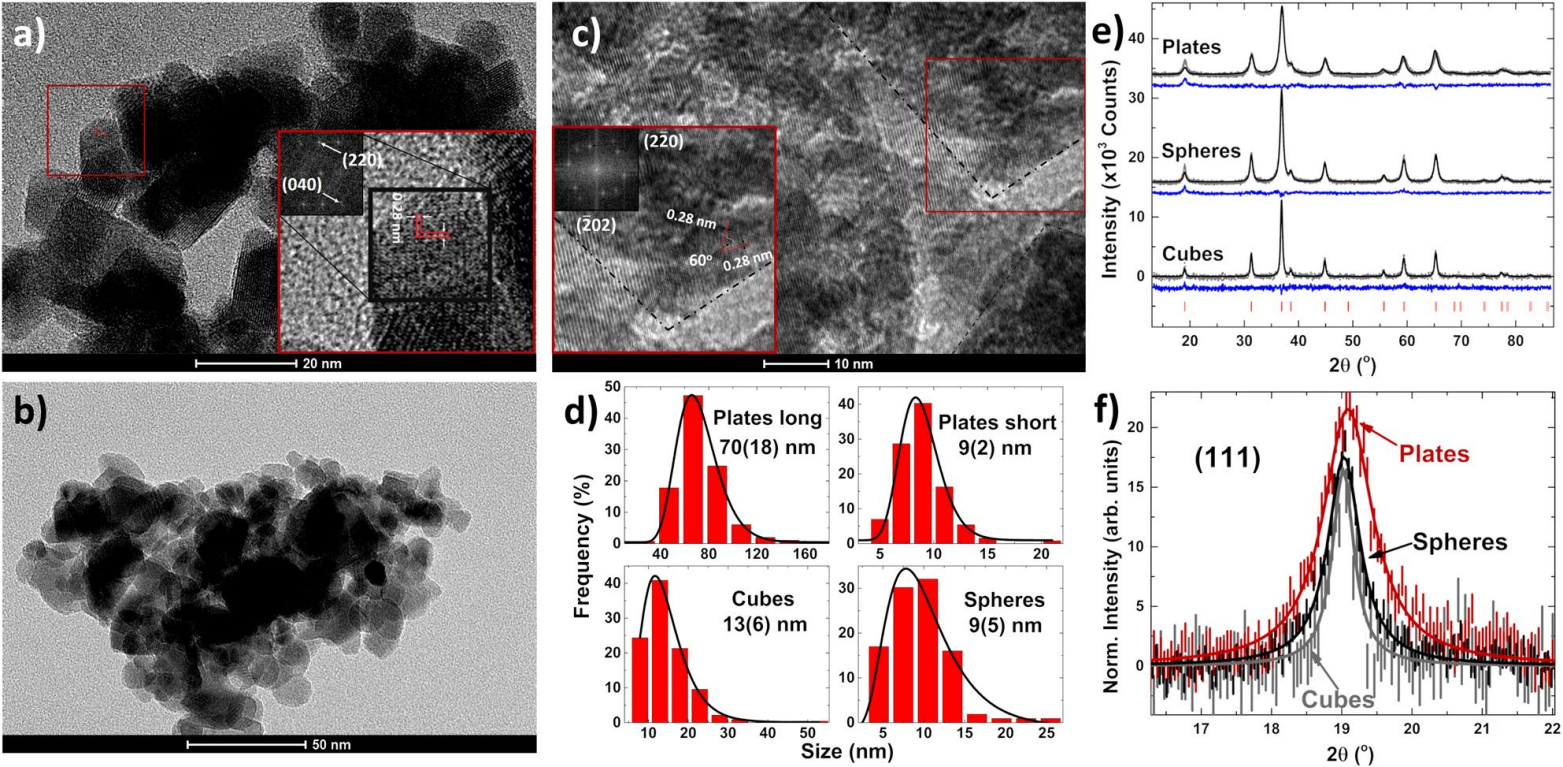
(**a**) TEM image of the cubes. The inset shows the planes at a 90° angle to one another and the fast fourier transform (FFT). (**b**) TEM image of the spheres is shown. (**c**) TEM image of the plates. The inset shows the planes at a 60° angle to one another and their FFT. (**d**) Size distributions of the different shapes from both TEM and SEM images. (**e**) XRD patterns for all three of the nanoshapes with residuals (blue lines) and peak markers (red ticks) from Rietveld refinement. (**f**) Lorentzian fits of the XRD intensities over the (111) reflection as discussed in the text.

Typical transmission electron microscopy (TEM) images of the nanoshapes are shown in Fig. 1a–c. The spheres and cubes have an average diameter and side length of 10 and 15 nm, respectively. The hexagonal plates are 70 nm across with a thickness of 8 nm. The visible lattice planes for the cubes are the (022) and (0$\bar{2}$2) planes (Fig. 1a) occurring with a 90° angle between them, with a distance of 0.28 nm between successive planes. These planes are perpendicular to the {100} family of planes exposed at the surface. The plates show the (2$\bar{2}$0) and ($\bar{2}$02) planes 60° apart, with a separation of 0.28 nm between different planes (Fig.  1c), perpendicular to the {111} family. From TEM we can see that the plates are textured with domains with sizes of roughly 7–8 nm.

It has been established from surveys of shapes that cubes predominantly expose the (100) planes while (hexagonal) plates expose the (111) planes^1,9–12^. The size distributions of the different shapes have lognormal distributions with the mean sizes and standard deviations reported in Fig. 1d). The size distribution for the plates was obtained from scanning electron microscopy (SEM) images while additional TEM images for the spheres and cubes are found in the supplemental information (SI).

X-ray diffraction results are presented in Fig. 1e). Rietveld refinements were done with GSAS-II^13^. The patterns were all phase pure $\hbox{Co}_{3}\hbox{O}_{4}$ with the expected Fd$\bar{3}$m space group. Occupancies were unchanged between the different shapes, indicating that the structure is the anticipated normal spinel. The crystallite sizes obtained from refinements match those from TEM image analysis; plates are composed of many crystallites (9(1) nm) while the cubes (25(1) nm) and spheres (15(1) nm) are single nanocrystallites. All lattice constants (spheres *a*=8.075(3) Å, cubes *a*=8.078(3) Å and plates *a*=8.062(3) Å) are close to the bulk value of *a* = 8.065(3) Å^2^. Preferred orientation was found for the the plates along the (111) reflection. Since preferred orientation is due to surface planes, the magnitude is small, and is most easily shown with normalized intensities for the (111) plane (Fig. 1f). The data most clearly shows the difference in intensity caused by preferred orientation for the (111) plane, with no other reflection displaying this difference (other than (222), etc.). Solid lines are Lorentzian curves to guide the eye for each shape over the (111) reflection, where the plates show an increase of roughly 25% in normalized intensity over the other shapes due to preferred orientation.

### Surface environment and cation distribution

To obtain information about the different surface terminations for the shapes, we can use a program to reconstruct the different exposed planes. Wherein the surface terminations can elucidate the magnetic behaviour caused by the different shapes. Visualization of the shapes with their surface planes was performed using VESTA^14^ to quantify the total surface areas, the surface areas of planes, and the Co$^{2+}$ to Co$^{3+}$ ratios of those planes. The Co$^{2+}$:Co$^{3+}$ ratio found at the surface of the shapes are 1.8:2 for the plates, 1.4:2 for the spheres, and the cubes matching the Co$^{2+}$:Co$^{3+}$ ratio from bulk $\hbox{Co}_{3}\hbox{O}_{4}$ (1:2). For the spherical particles there are no specific exposed planes, but an average of equal amounts of three different families of $\left\{ 100\right\}$, $\left\{ 110\right\}$, and $\left\{ 111\right\}$ planes, which gives 26 exposed planes; six from the $\left\{ 100\right\}$, 12 from the $\left\{ 110\right\}$, and eight from the $\left\{ 111\right\}$ family. As a way to correlate certain properties to the different shapes, we look at the surface area of the (111) plane for the different shapes. This, along with the total calculated surface areas (m$^{2}$/g) and the Co$^{2+}$:Co$^{3+}$ ratio at the surface for the different shapes are shown in Table 1.

**Table 1:** $\hbox{Co}_{3}\hbox{O}_{4}$ exposed planes.

Shape (Plane)	Surface area (m$^{2}$/g)	(111) Surface area (m$^{2}$/g)	Co$^{2+}$:Co$^{3+}$ surface ratio
Spheres (100), (110), (111)	110.0	33.9	1.37:2
Cubes (100)	58.5	0.0	1:2
Plates (111)	25.0	20.2	1.75:2

**Figure 2:**
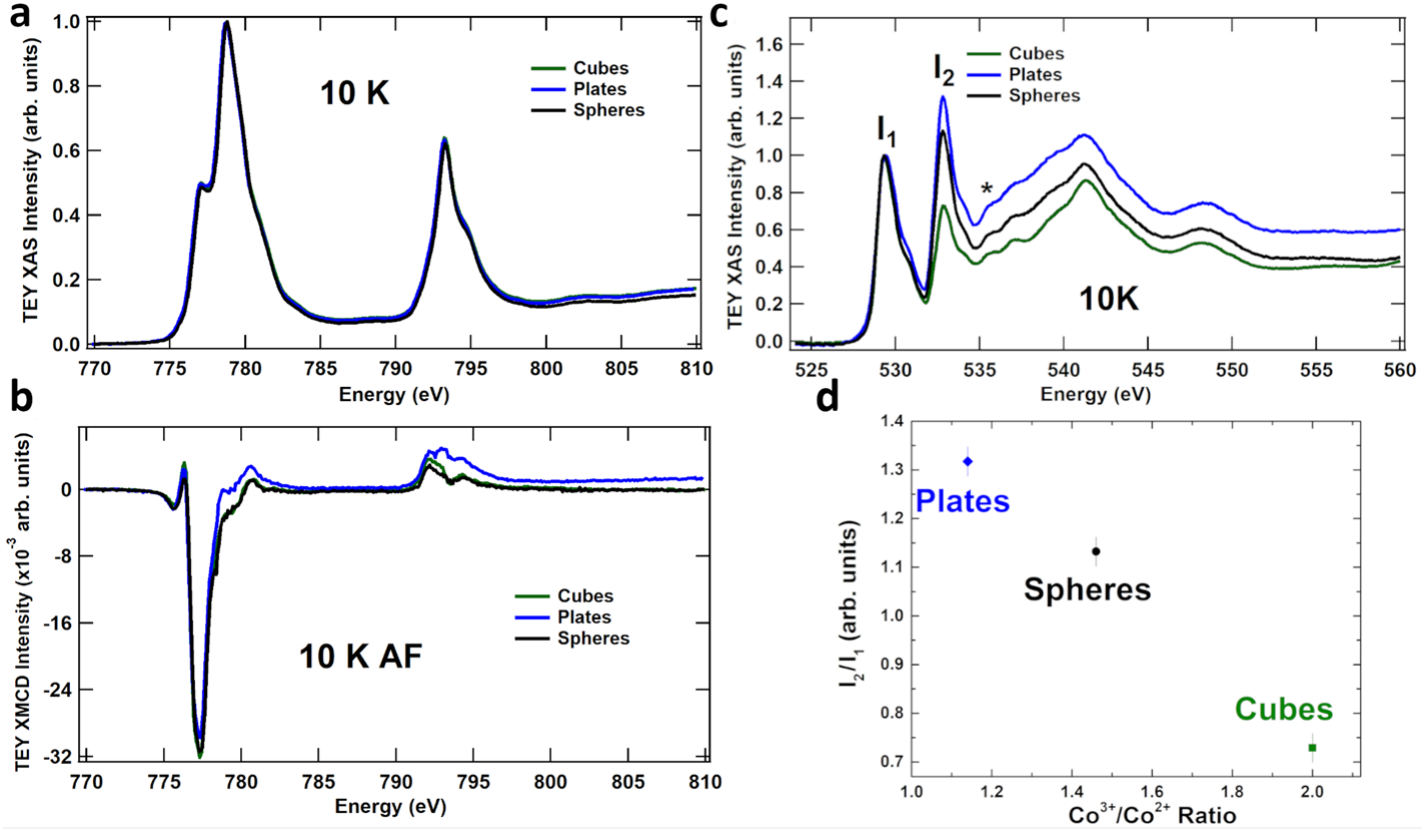
(**a**) Co $\hbox{L}_{3,2}$-edge XAS and (**b**) XAS normalized XMCD spectra at 10 K, obtained under applied fields of 50 kOe using TEY detection. (**c**) O K-edge XAS spectra at 10 K. The $t_{2g}$ and $e_g$ peaks are labelled $I_1$ and $I_2$, respectively; the asterisk marks the peak from the oxygen on the carbon tape^15^. (**d**) The variation of $I_2/I_1$ between the shapes is shown. Indicating a difference in occupation of the Co$^{3+}$ ions at the surface.

XAS characterizes the partial density of states just above the occupied density of states (Fermi energy) that can be influenced by hybridization with the ions’ ligand orbitals, thus identifying the different species of ions present, their coordinations and vacancies. In perovskite structures, oxygen vacancies can lead to a change in the lattice parameter. The vacancies promote charge transfer resulting in a measurable change in oxidation state for the cations^16^. Figure 2a shows the Co $\hbox{L}_{3,2}$-edge spectra for each of the shapes, where the large peak at 779 eV corresponds to Co$^{3+}$ and the shoulder at 777 eV corresponds to Co$^{2+}$. All spectra are essentially the same for the shapes and describe a Co$^{2+}$:Co$^{3+}$ ratio of 1:2. Co L-edge XAS reveals no change to the cation occupations. Thus, for the different shapes, we find no change that would be caused by oxygen vacancies in the structure. Keep in mind that while TEY is a surface probe (2–3 nm probe depth for these $\hbox{L}_{3,2}$ energies^17^, we would expect to see at most an intensity change of 2% between the spheres and plates (see SI).

The O K-edge XAS for the different shapes presented in Fig. 2c quantifies the hybridization of the metal Co$^{3+}$ 3*d* orbitals with the O$^{2-}$ ligand 2*p* orbitals via splitting of the major (spin-up) and minor (spin-down) $e_{g}$ and $t_{2g}$ energies in addition to crystal field splitting effects^18^. The observed changes in peak intensities at 530 and 533 eV provides a measure of the available 3*d* hole states on the Co ions hybridized with the O ions, while energies above 535 eV indicate hybridization with 4*s* orbitals. Quantitatively, the plates have a higher number of available $e_{g}$/$t_{2g}$ states on the Co$^{3+}$ caused by a concomitant decrease in the coordination at the surface^1,18^. O K edge measurements provide a great link between the different surface terminations for the shapes through the different bonding environments of the oxygen ions. Correlating the ratios of the $e_g$/$t_{2g}$ peaks, along with surface areas can provide us with the number of ions and coordinations present at the surface.

The variation of $\hbox{I}_{2}$/$\hbox{I}_{1}$ vs Co$^{3+}$/Co$^{2+}$ ratio shown in Fig. 2d clearly identifies the differences between the spheres, cubes, and plates from the majority of oxygen ions at the surfaces; directly related to the $\text{ Co }{{-}}\text{ O }$ bond combinations for the different shapes, following the Co$^{2+}$:Co$^{3+}$ surface ratios identified using VESTA. At the surface of the cubes the (100) plane contains Co$^{3+}$ ions coordinated to five oxygen ions ($\hbox{CoO}_5$), while the (111) plane contains three fold coordination on the exposed Co$^{3+}$ ion ($\hbox{CoO}_3$). From cubes, to spheres, to plates there are changes in the O 2*p*-to-Co 4*s* hybridization. This is the result of the exchange paths of the exposed planes resulting in changes to the $\text{ Co }{{-}}\text{ O }$ bonding between the different shapes, affecting the measured hybridization. The cubes show more defined peaks due to hybridization from 537 to 545 eV (consistent with highly coordinated transition metal ions), indicating the stability of the (100) planes at the surface of the cubes^1^.

Probing only the magnetic ions, ferromagnetism is most cleanly observed via X-ray magnetic circular dichroism (XMCD). Using both right and left circularly polarized X-rays to measure both spin-up and spin-down populations, an XMCD spectrum is not observable for antiferromagnets. For $\hbox{Co}_{3}\hbox{O}_{4}$, crystal field splitting dictates that all spins should be paired in the lower $t_{2g}$ orbital of the Co$^{3+}$ ions, while Co$^{2+}$ contains three unpaired spins ($S=3/2$) in the $t_{2g}$ orbital. The XMCD spectra in Fig.  2b collected over the Co $\hbox{L}_{3,2}$-edge for all three nanoshapes provide irrefutable evidence of ferromagnetic behaviour—an antiferromagnet with equal spin up and down populations presents no XMCD signal. We find that 30–50% of the signal is due to the surface ions on the shapes. It should be noted that an XMCD signal occurs with the surface-probe total electron yield (TEY) measurements; total fluorescence yield (TFY) spectra that are bulk sensitive show a vastly decreased XMCD signal (for further discussion, see SI). The differences between the shapes’ XMCD spectra over the $\hbox{L}_{3}$ edge at 779 eV are due to the different Co$^{3+}$ environments at those surfaces. A decrease in the Co$^{3+}$ coordination at the surface causes a change in the crystal field environments resulting in an additional magnetic moment on the Co$^{3+}$ ions of the (111) plane. This can be further identified through the overall magnetic properties.

### Magnetism of the surface terminations

**Figure 3:**
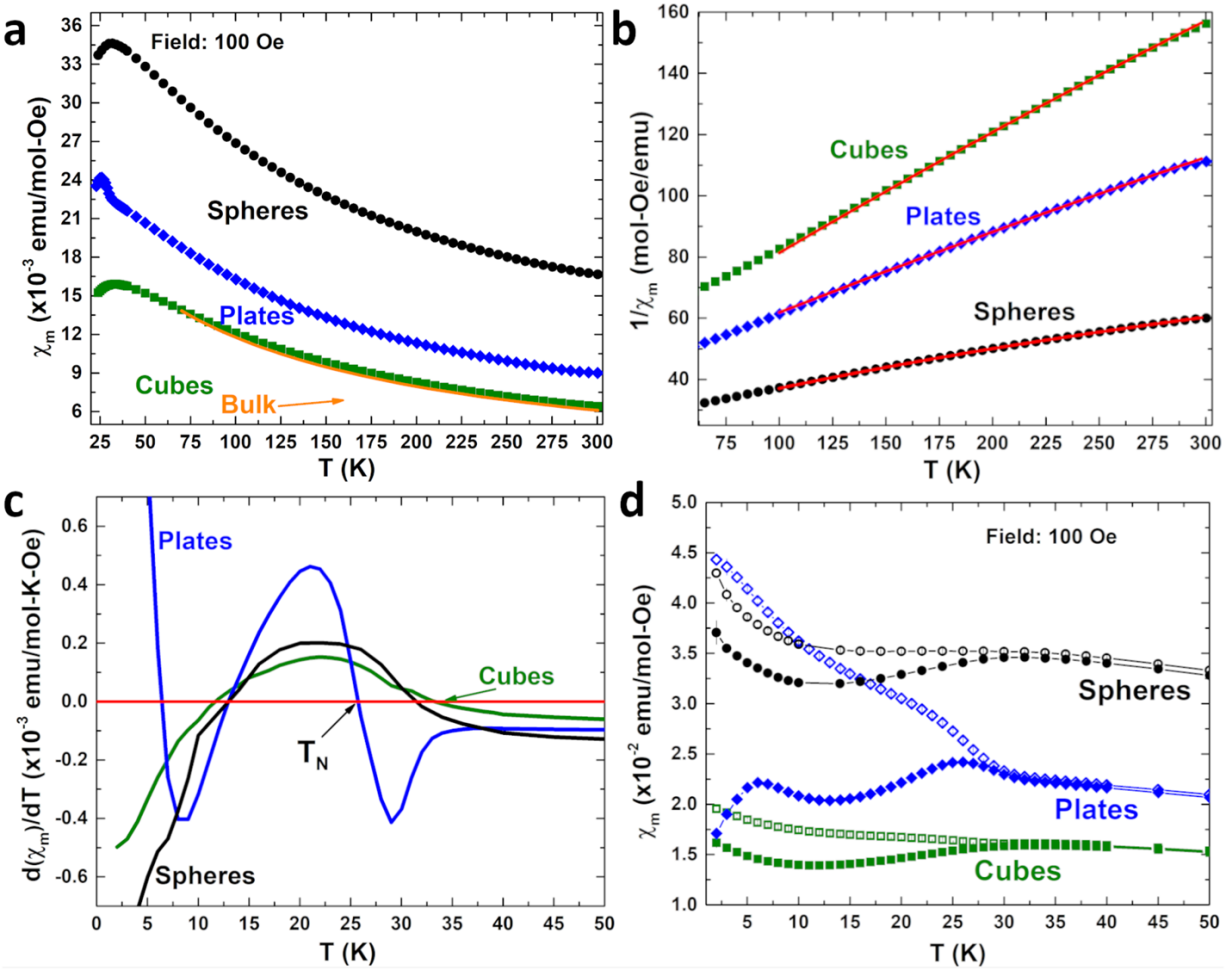
(**a**) Molar susceptibility ($\chi _m$) of the shapes from 20 to 300 K with the susceptibility of bulk shown in orange. (**b**) Fits (red lines) of the inverse susceptibility $1/\chi _m(T)$ in the region of 170–280 K. (**c**) Derivative $d\chi _m/dT$ shows the Néel temperatures for the different shapes. (**d**) Zero field cooled ($\chi _{ZFC}(T)$, closed symbols) and field cooled ($\chi _{FC}(T)$, open symbols) susceptibility of the $\hbox{Co}_{3}\hbox{O}_{4}$ nanoshapes.

We can quantify the differences in the Co ions’ surroundings (bonding, exchange interactions) on the different surface terminations of the nanoshapes by the straight-forward differences in the overall magnetism. For example, the different shapes’ low-field DC susceptibilities ($\chi _{DC}(T)$) in Fig.  3a gives the ordering temperature of each shape and the magnetic moment per formula unit—allowing the quantification of the (overall) superexchange present in each shape. Keep in mind that bulk $\hbox{Co}_{3}\hbox{O}_{4}$ is antiferromagnetic with $T_N=40$ K; the shapes show variations in $T_{N}$ where the spheres and cubes have $T_N$’s of 31(1) and 33(1) K, respectively, while the plates have $T_N$=25(1) K (The Néel temperatures were take from the peak in $\chi _{DC}$ and when $d\chi _{DC}/dT$=0, Fig. 3c. Quantification of the exchange and the magnetic moments were from analysis of $1/\chi _{DC}(T)$ (Fig. 3b) above the Néel temperature, where in this temperature regime the systems are all paramagnetic so $\chi _{DC}(T)$ can be represented by the well known modified Curie–Weiss Law $\chi _{DC}(T)=\chi _{0}+\frac{C}{T-\theta }$, where $\chi _{0}$ is a temperature independent component to the susceptibility (Van Vleck paramagnetism), *C* is the Curie constant, and $\theta$ is the Weiss temperature (for an antiferromagnet $\theta <0$). The results of the fits are in Table 2.

We can now quantify any differences from bulk $\hbox{Co}_{3}\hbox{O}_{4}$ and the variations betwen the samples related to the surface areas and exposed planes. Since the temperature independent susceptibility, $\chi _{0}$, of bulk $\hbox{Co}_{3}\hbox{O}_{4}$ was shown to have near ionic-like values of Van Vleck paramagnetism for Co$^{2+}$ and Co$^{3+}$ ions^19^, we would expect $\chi _0$ to remain little affected by the size and shapes. Indeed, all three shapes have a $\chi _{0}$ essentially identical to the bulk value. $\theta$, the Weiss temperature, is related to the exchange interaction strength (bulk values for $\theta$ are reported between − 53 and − 110 K^2,20–22^), and all three shapes have $\theta$ values consistent with the bulk values. $\mu _{eff}$ gives a quantitative measure of the overall magnetic moment ($\mu _{eff} = 2.83{\times }C^{1/2}$), and there is an increase in the Curie constant *C* (and $\mu _{eff}$) that depends on the shape of the particle (Table 2). *C* is largest for the spheres due to the highest overall surface area, followed by plates and cubes. But, even though the cubes have a larger per particle surface area than the plates, their magnetic moment is near bulk. The spheres expose the (100), (110), and (111) planes equally, however the plates expose the surface (111) planes, and the increase in *C* can be related to this plane. The change in the overall Co moment of the nanoparticle between the different shapes is related to the surface coordination and configuration of the ions on those planes.

**Table 2:** Magnetic properties.

Shape	$\chi _0$ (emu/mol Oe$^{-1}$)	C (emu K/mol Oe$^{-1}$)	$\theta$ (K)	$\mu _{eff}$ ($\mu _{B}$)	$J_{ij}$ (K/$\hbox{k}_B$)
Spheres	$7(3){\times }10^{-3}$	4.0(1)	− 93(5)	5.7(1)	1.5(2)
Plates	$1(1){\times }10^{-3}$	3.1(1)	− 107(5)	5.0(1)	1.6(1)
Cubes	$1(1){\times }10^{-3}$	2.1(5)	− 83(5)	4.1(5)	3.0(1)
Bulk^22–22^	$0.7{\times }10^{-3}$	2.1	− 85	4.1	4.0

Furthermore, $\mu _{eff}$ and $T_N$ from the DC susceptibility provide a measure of the exchange interaction strengths from the surface. Since between magnetic ions $\mathbf {S}_{i}$ and $\mathbf {S}_{j}$ exchange is described by the Hamiltonian, $H_{ex} = -2J_{ij}\mathbf {S}_{i} \cdot \mathbf {S}_{j}$ where $J_{ij}=\frac{3k_{B}T_{N}}{2zJ(J+1)}$, and *z* is the number of nearest neighbours. For bulk $\hbox{Co}_{3}\hbox{O}_{4}$ with $T_{N} = 40~\text{ K }$, $z = 4$ and $J = S$ (the spin only value with $S = \frac{3}{2}$) we find $J_{ij} = 4.0~\text{ K }/k_{B}$. Due to a large crystal field splitting and small atomic number, the 3d transition metal Co ions have quenched orbital angular momentum ($L=0$). For the different shapes we do not use the spin-only value for $J(J+1)$, instead we obtain the total angular momentum from $\mu ^{2}_{eff}=4J(J+1)\mu ^{2}_{B}$ (obtained from magnetometry). This is to account for $\mu _{eff}$ shown to describe contributions from the surface spin magnetism^23^, where broken symmetry and low coordination provide mechanisms for the presence of unquenched angular momenta for surface atoms, and to account for the possibility of a small magnetic moment present on the Co$^{3+}$ ion^24^. The values for the exchange $J_{ij}$ for the shapes are presented in Table 2. The exchange in the cubes are most similar to that of the bulk. Overall, the plates have a $\mu _{eff}$ larger than bulk and a much lower $T_N$, indicating a weaker $J_{ij}$ (factor of two weaker), linked to the (111) plane exposure, and the spheres have the weakest exchange (largest overall moment) due to the combination of highest surface area and a (111) plane exposure (Table 1).

Low coordination on the Co$^{3+}$ ions and uncompensated surface ferromagnetism (as revealed by the Co and O XAS and XMCD) lead to a larger magnetic moment from the spins on the (111) planes. We can examine the magnetism below $T_{N}$ as a function of applied field and temperature, to further identify characteristic behaviour. Figure 3d shows the zero field cooled (ZFC, $\chi _{ZFC}(T)$) and field cooled (FC, $\chi _{FC}(T)$) susceptibilities for the shapes below 50 K, where the spheres and cubes display very similar behaviour in both ZFC and FC susceptibilities—an increase in $\chi _{ZFC}(T)$ and $\chi _{FC}(T)$ as temperatures cooled below 12 K. This is the result of a reconfiguration of the spins at the surface. It is characteristic behaviour seen in other systems displaying an antiferromagnetic spin-flop^6,25,26^. An antiferromagnetic spin-flop occurs at a critical value of the applied magnetic field in antiferromagnets where the spins parallel to the magnetic field undergo a (90°) reorientation producing a net magnetic moment in the direction of the applied field. For the spins in the shapes near the surface, a decrease in the strength of the exchange interactions cause the spin-flop to occur at a lower critical applied field than the core of the particles^5,7^. An illustration of a surface spin-flop is shown in the SI. By contrast, the nanoplate’s strong response with cooling of $\chi _{FC}(T)$ below $T_{N}$ is representative of ferro-/ferrimagnetism. The plates present a large bifurcation below $T_N$ revealing the dominant ferromagnetic interactions at the surface.

**Figure 4:**
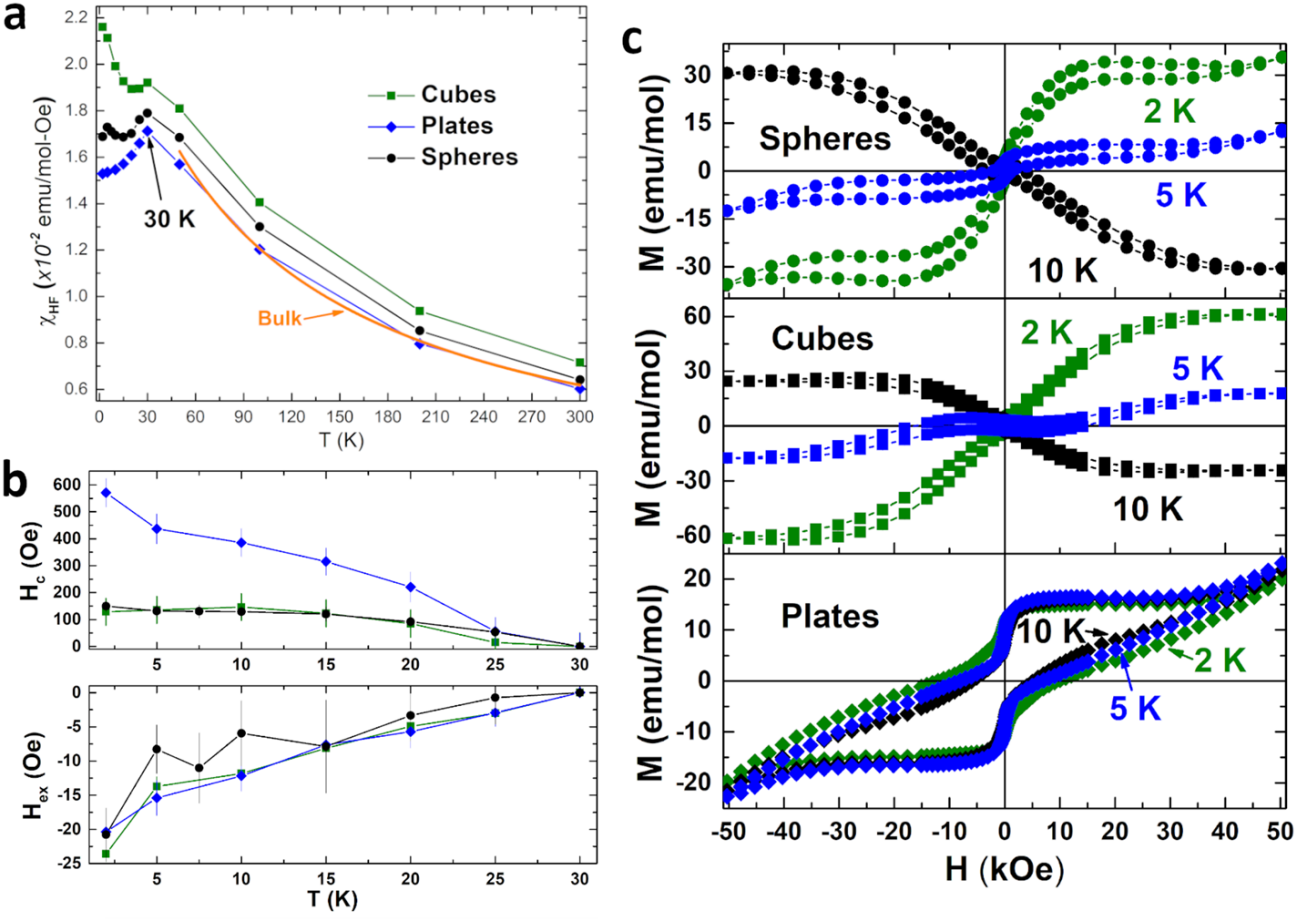
(**a**) $\chi _{HF}$ vs T is shown for all the shapes. The high-field susceptibility is from the core of the particles. For the plates, $\chi _{HF}$ peaks at 30 K and above 50 K all the shapes behave similar to bulk (orange). (**b**) Coercivity ($H_c$) and exchange bias ($H_{ex}$) below the ordering temperature $T_N$. (**c**) Surface component of the *M* versus *H* loops, obtained from subtraction of the high-field susceptibility.

The high-field susceptibility $\chi _{HF}$ is obtained by fitting a linear component above fields of $H > 30~\text{ kOe }$ in the *M*(*H*) data. The overall magnetization can be represented by an equation that describes all the contributions to the magnetization^27,28^: $M(H) = M_{surf}(H) + \chi _{HF} H$. Generally, the high-field susceptibility contains contributions from the bulk and the subtracted hysteresis loops, $M_{surf}(H)$, represent the remaining ferromagnetic contributions to the magnetization from the particles’ surface. Temperature dependencies of the high-field susceptibility $\chi _{HF}(T)$ in Fig.  4a identifies core behaviour for the samples and aids in assigning the remaining contributions to the different surface terminations. $\chi _{HF}(T)$ for all three shapes show values of the magnetic moment and exchange constant nearly identical to the bulk $\chi _{DC}(T)$. Thus, the core of each of the shapes can be described by bulk $\hbox{Co}_3\hbox{O}_4$, lending credence to our interpretation that the surface magnetism dominates the low-field susceptibility. Additionally, we find that $T_N$ for the plates in $\chi _{HF}(T)$ peaks at 30 K, different from $\chi _{DC}(T)$ and closer to the bulk value. This is further evidence that the core orders differently from the surface.

The hysteresis loops, $M_{surf}(H)$, describing the surface are shown in Fig. 4c are obtained by subtracting $\chi _{HF} H$ from the *M*(*H*) data using the above equation^27,28^. The spheres and cubes display “inverted” hysteresis loops at temperatures between 10 and 25 K, the clearest indication that a spin-flop transition has occurred, and surface antiferromagnetic interactions are still dominant^5–7,25,26^. As temperature decreases, the thermal activation energy decreases leading to stronger competition at the surface layer. This lowers the field at which the spin-flop can occur. By 2 K, the spin-flop occurs at such a small field that the magnetization of the surface (spin-flop) layer saturates.

It has been long established^29^, that when two adjacent ferro- and antiferromagnetic systems are coupled an exchange occurs between the two materials known as exchange bias. The exchange between the materials can result in enhanced coercivities, horizontal, and vertical loop shifts. For the shapes, the presence of exchange bias reveals the existence of distinct populations of spins at the surface. The surface spins (the spin-flop layer of the spheres and cubes and the ferromagnetic layer of the hexagonal plates) couple to the antiferromagnetic core enabling a coercivity ($H_c$, in an overall antiferromagnet), a vertical loop shift ($\Delta M_s$) and exchange bias ($H_{ex}$). $H_c(T)$ and $H_{ex}(T)$ are shown for all of the shapes in Fig.  4b. The magnetization for the plates show ferromagnetic behaviour, the with magnetic saturation remaining positive and constant throughout the temperature region below $T_N$. The plates have a larger (4–5×) coercivity than the other shapes that also remains relatively constant, indicating the strong ferromagnetic interactions and anisotropy occurring from the surface Co ions (Fig. 4b). The surface of the plates cannot be described by spin-glass behaviour. As the surface spins freeze we would expect to see a large increase in the coercivity and exchange bias field^30,31^. The plates show a constant coercivity at temperatures below $T_N$, and all shapes show similar behaviour in $H_{ex}$ indicating that no freezing is occurring at the surface^32–34^.

## Discussion

**Figure 5:**
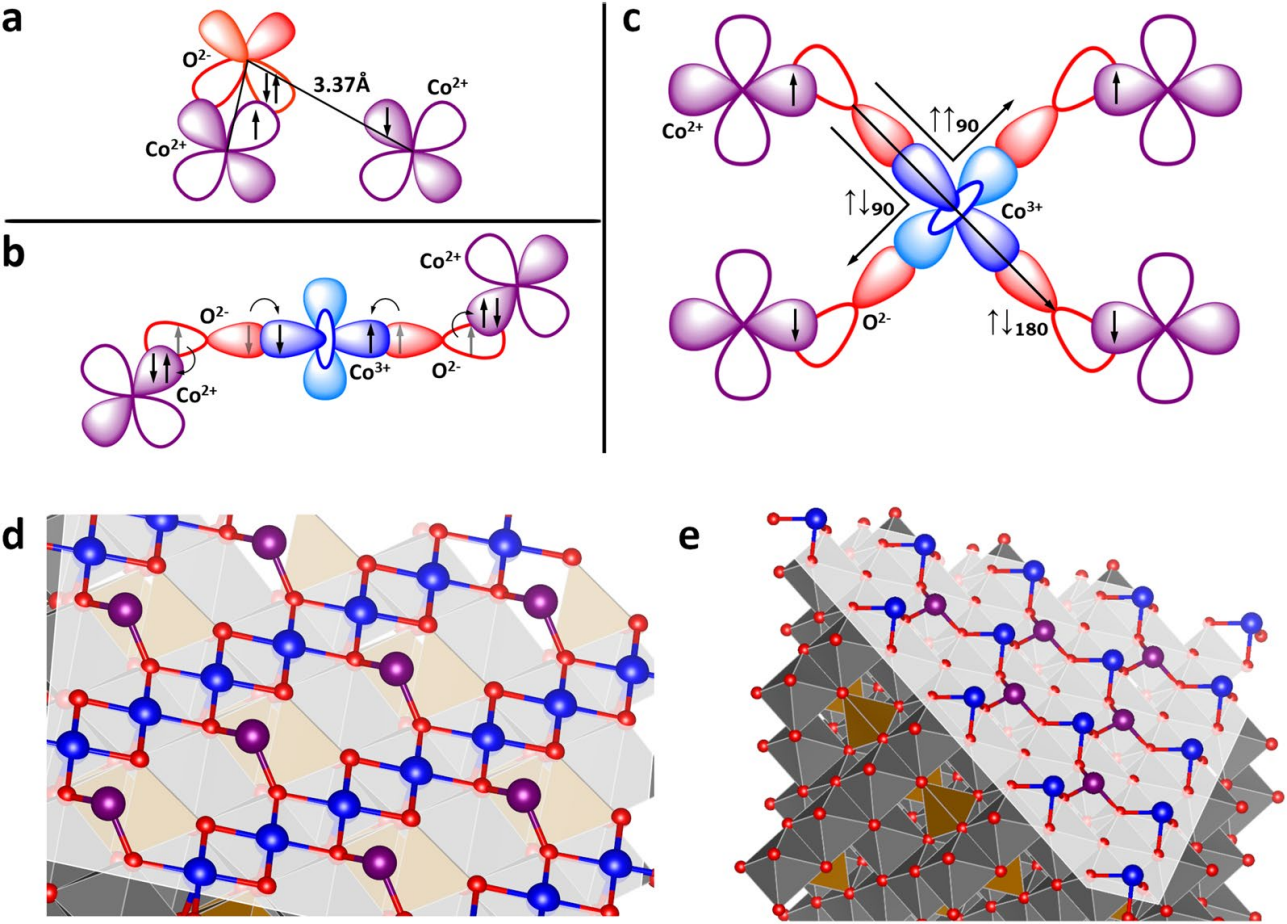
(**a**) Shows the interaction path of the ${\text{Co}}^{{2+}} {{-}}{\text{O}}{{-}}{\text{Co}}^{{2+}}$ configuration. O$^{2-}$ orbitals are shown in red and orange; Co$^{2+}$ and Co$^{3+}$ orbitals are shown in purple and blue, respectively. (**b**) Shows one of the possible configurations for the ${\text{Co}}^{{2+}} {{-}}{\text{O}}{{-}}{\text{Co}}^{{3+}} {{-}}{\text{O}}{{-}}{\text{Co}}^{{2+}}$ pathway. (**c**) Shows all of the extended ${\text{Co}}^{{2+}} {{-}}{\text{O}}{{-}}{\text{Co}}^{{3+}} {{-}}{\text{O}}{{-}}{\text{Co}}^{{2+}}$ exchange pathways. (**d**) Illustration of the (100) and (**e**)  (111) surface terminations for the cubes and plates, respectively.

The superexchange interaction is the dominant mechanism for exchange between the magnetic Co$^{2+}$ ions in $\hbox{Co}_{3}\hbox{O}_{4}$. The different exchange mechanisms present are shown in Fig. 5a–c). Where the more direct Co$^{2+}$–O–Co$^{2+}$ (Fig. 5a) superexchange interaction only accounts for a small ($\sim$10%) portion of the total exchange strength. The three different $\text{Co}^{2+}{{-}}\text{O}{{-}}\text{Co}^{3+}{{-}}\text{O}{{-}}\text{Co}^{2+}$ paths are the reason for the strong antiferromagnetic order in the bulk. The virtual electron exchange interactions that occur in the superexchange interaction manifest in the extended interaction (Fig. 5b). Although the exchange interactions are extended over three intervening ions, the general rules for superexchange can be applied to $\hbox{Co}_{3}\hbox{O}_{4}$, with one main distinction: The differences in the exchange paths in $\hbox{Co}_{3}\hbox{O}_{4}$ come from the $\text{O}{{-}}\text{Co}^{3+} {{-}}\text{O}$ bond rather than the usual metal–oxygen–metal configuration^35–37^. Using the convention of $\uparrow \downarrow _{180}$, $\uparrow \downarrow _{90}$ and, $\uparrow \uparrow _{90}$ for the three different superexchange paths where the arrows indicate the interaction is either antiferromagnetic ($\uparrow \downarrow$) or ferromagnetic ($\uparrow \uparrow$) and the subscript denotes the $\text{O}{{-}}\text{Co}^{3+} {{-}}\text{O}$ bond angle.

The three different exchange paths are shown in Fig.  5c). $\uparrow \downarrow _{180}$ is antiferromagnetic due to the coupling of the O$^{2-}$ ions through the same Co$^{3+}$ orbital. For the two 90° exchange paths, one is ferromagnetic ($\uparrow \uparrow _{90}$) due to the coupling through separate Co$^{3+}$ orbitals, and one is antiferromagnetic ($\uparrow \downarrow _{90}$) because the two interactions are always 180° to each other through the same Co$^{3+}$ orbital (Fig. 5c)^35–37^. This leads to opposing interactions from the Pauli exclusion principle. With competing ferro- and antiferromagnetic interactions present at the surface there is the potential for weakened antiferromagnetic or dominant ferromagnetic interactions. Paths $\uparrow \downarrow _{90}$ and $\uparrow \uparrow _{90}$ have the same bond distances, angles, and multiplicities. To first order, the interaction strengths cancel^2^. Thus, path $\uparrow \downarrow _{180}$ is then the only uncompensated interaction path and is responsible for the strong antiferromagnetic exchange in bulk $\hbox{Co}_{3}\hbox{O}_{4}$. At the surface, we can quantify this competition between $\uparrow \downarrow _{90}$ and $\uparrow \uparrow _{90}$ exchange paths, using the multiplicities for exchange paths given in Table 3 we formulate the quantity $\delta$ given by: $\delta = \frac{\left[ 1+ (M_{\uparrow \uparrow _{90}} - M_{\uparrow \downarrow _{90}})\right] }{M_{\uparrow \downarrow _{180}}}$, where *M* is the multiplicity of the path given in the subscript, and we find that a $\delta > 1.1$ characterizes dominant ferromagnetic interactions at the surface, $\delta < 1.1$ characterizes dominant antiferromagnetic interactions at the surface, and $\delta = 1/12$ is the value for bulk $\hbox{Co}_3\hbox{O}_4$. For the different shapes we find that the cubes have a value of $\delta _{100}=0.83$, the plates have a value of $\delta _{111}=2.17$, and the spheres have a value of $\delta _{\mathrm{sphere}}=0.92$ (obtained from an average of $\delta _{hkl}$ for the three families of planes at the surface of the spheres). These three values for $\delta$ are in agreement with the analysis presented for the magnetic structure shown above, where the spheres are shown to display antiferromagnetic behaviour similar to the cubes, despite the large exposure of the (111) plane similar to the plates.

**Table 3:** $\hbox{Co}_{3}\hbox{O}_{4}$ surface exchange pathways.

Multiplicity	(111) Plane	(110) Plane	(100) Plane	Bulk
$\uparrow \downarrow _{180}$	6	7	6	12
$\uparrow \downarrow _{90}$	6	12	12	24
$\uparrow \uparrow _{90}$	18	12	16	24

Visualizations from VESTA of the surface of the (100) plane at the surface of the cubes, and (111) plane at the surface of the plates, are shown in Fig. 5d,e, respectively. The (100) plane shows the formation of the Co$^{3+}$–O planes with a much higher Co$^{3+}$ coordination leading to the stability of the (100) plane.

The nature of the (111) plane is very different, with Co$^{3+}$ ions threefold coordinated to oxygen ions in a zig–zag like structure, where the Co$^{3+}$ ions directly at the surface can only promote the ‘W-shaped’ $\uparrow \uparrow _{90}$ exchange path. Examining the surfaces for all three planes—(100), (110), and (111), the exchange interactions present are shown in Table 3, where the multiplicity is the total number of possible interaction pathways from a Co$^{2+}$ ion to all neighbours of a given type (e.g. nearest or next-nearest neighbours). We can now begin to examine the impact of the surface ions of the different terminations on the overall magnetic properties.

Examining the magnetic behaviour for the cubes, we find the (100) plane displays a much stronger antiferromagnetic exchange relative to the (111) plane of the plates. This is a result of the configuration of the Co$^{2+}$ and Co$^{3+}$ ions at the different surfaces that was found through the structural and magnetic properties, such as the effective magnetic moment of the different planes and the direct variation in bonding observed from XAS and XMCD. The O K edge XAS provides us with a direct link between the magnetic properties that arise from the bonding between the shapes. With the ability to describe the coordination and environments for the Co ions, this allows us to correlate the magnetic properties for the different planes and shapes.

Broken exchange pathways at surfaces result in significant changes to the Co$^{2+}$:Co$^{3+}$ ratios and the local coordination, as we have quantified. This changes the local exchange structure of the surface enabling interesting magnetic behaviour such as an antiferromagnetic spin-flop and distinct ferromagnetism. We found that only the (111) plane contains Co$^{3+}$ with a magnetic moment corresponding to the low coordination of Co$^{3+}$ ions (3/6) and dominant ferromagnetic exchange. The coordination of Co$^{3+}$ ions on the (100) and (110) planes are closer to bulk (5/6 and 4/6, respectively)^1^. The quantity $\delta$ provides a good measure of the interactions at the surface—but these interactions are based on the available coordination for the ions. Thus, we find that $\delta$ is than related to the overall coordination of the ions at the surface (through exchange interactions).

Individually, the cubes and plates allow us to characterize the magnetic behaviour for both the (100) and (111) plane, respectively. The weakening of the antiferromagnetic exchange paths at the surface of the plates is much greater. The cubes contain a larger surface area over the plates, but still display hindered magnetic behaviour. The surface of the cubes contain a highly coordinated plane of Co$^{3+}$ and O ions, promoting antiferromagnetic interactions, but display a spin-flop showing ferromagnetic-like behaviour due to the coupling with the antiferromagnetic core. The surface of the plates contains an additional magnetic moment arising from the Co$^{3+}$ ions. Low coordination alters the crystal field environment increasing the anisotropy of the surface and allowing for the appearance of a magnetic moment. The dominant ferromagnetic interactions of the (111) plane promote ferromagnetism at the surface of the plates.

Through XMCD and magnetometry, it was found that the core of each shape is well described by bulk $\hbox{Co}_3\hbox{O}_4$. As an antiferromagnet, the absence of a net magnetic moment in the core precludes the possibility of shape anisotropy^38^. Thus, any increase in the effective anisotropy comes from the different surface environments such as steps, kinks, and exchange interactions^38,39^. Examining the (111) plane (Fig. 5e), we find that the ions at the surface align in a stepped configuration which greatly increases the anisotropy of a surface. In addition, there is a complementary increase in anisotropy introduced by the exchange interactions involving the lowly-coordinated Co$^{3+}$ ion^39^. This results in the increased coercivity that was obtained for the hexagonal plates. The plates have the smallest overall surface area, so the large coercivity reveals that the anisotropy of the (111) plane is greatly enhanced over the other planes.

For the spheres the magnetic properties can be shown to be a combination of the different exposed planes, where the (100), and (111) plane were characterized using cubes and plates, respectively. The exchange constant $J_{ij}$ for the spheres is the smallest, with the extra contribution of the effective magnetic moment arising from the exposed (111) plane. Based on surface area arguments the value for the effective magnetic moment, and thus, exchange constant $J_{ij}$ for the spheres can be correlated to the exposed planes of the surface, as described above. We were also able to characterize the overall magnetic behaviour with the parameter $\delta$ that is correlated to the surface exchange and coordination of the ions.

## Methods

### Synthesis

For the spheres, $\epsilon$-Co was synthesized by first dissolving $0.1~\text{ g }$ of trioctylphosphine oxide (TOPO) into $12~\text{ ml }$ of o-dichlorobenzene and $0.2~\text{ ml }$ of 99% oleic acid. The solution is then heated to 180 °C under an argon atmosphere. In a separate solution, 0.54 g of dicobalt octacarbonyl ($\hbox{Co}_{2}(\hbox{CO})_{8}$) was dissolved into 3 ml of o-dichlorobenzene (DCB) and injected into the heated (180 °C) solution of TOPO, oleic acid, and DCB. The obtained Co was washed using ethanol. The precipitate was dried in a furnace at 60 °C for 12 h in air. To obtain $\hbox{Co}_{3}\hbox{O}_{4}$ the $\epsilon$-Co was placed in a tube furnace with a initial heating rate of 5 °C/min until 300 °C was reached, and the temperature was held for 3 h. Subsequently, the temperature was ramped down at a rate of 2 °C/min. Oxidation of Co causes transformations to first CoO then finally $\hbox{Co}_{3}\hbox{O}_{4}$^40^.

For the cubes, a blue/green precipitate of cobalt hydroxide ($\alpha -\hbox{Co}(\hbox{OH})_{2}$) was synthesized by dissolving $9.52~\text{ g }$ of cobalt chloride $\hbox{CoCl}_{2}\cdot 6\hbox{H}_{2}\hbox{O}$ into $100~\text{ ml }$ of distilled (DI) water. $25~\text{ ml }$ of ammonium hydroxide $\hbox{NH}_{4}\hbox{OH}$ was added and the solution was stirred for two hours. Drying and calcination procedures were the same as the spheres.

For the plates, $\beta -\hbox{Co}(\hbox{OH})_{2}$ was similarly synthesized, dissolving $4.76~\text{ g }$ of $\hbox{CoCl}_{2}\cdot 6\hbox{H}_{2}\hbox{O}$ into $100~\text{ ml }$ of DI water, while $3~\text{ g }$ of sodium hydroxide (NaOH) is dissolved in $25~\text{ ml }$ of DI water. The hydroxide solution was added to a heated $\hbox{CoCl}_{2}$ solution (80 °C) and was stirred for two hours at 80 °C. Drying and calcination procedures were the same as the cubes and spheres above. $\beta$-$\hbox{Co}(\hbox{OH})_{2}$ contains divalent Co ions octahedrally coordinated to hydroxyl ions in a layered hexagonal brucite structure^41^. $\alpha$-$\hbox{Co}(\hbox{OH})_{2}$ forms a layered hexagonal hydrotalcite-like structure, but with Co ions in both tetrahedral and octahedral sites^41^. For the synthesis of the plates, the initial colour of the precipitate starts blue/green as an $\alpha$-$\hbox{Co}(\hbox{OH})_{2}$ but immediately changes to the characteristic pink colour of $\beta$-$\hbox{Co}(\hbox{OH})_{2}$. This occurs as the $\beta$ phase is the more thermodynamically stable of the two forms^42^. Over the course of the two hour synthesis the colour gradually changes from pink to a light brown. This indicates a transformation at the surface of the plates from $\beta$-$\hbox{Co}(\hbox{OH})_{2}$ to $\hbox{CoOOH}$ (cobalt oxyhydroxide) which contains trivalent Co ions. The plate shape is formed at the $\beta$-$\hbox{Co}(\hbox{OH})_{2}$ stage, after calcination the shape is retained with crystallites that form to compensate for the structural change.

The $\alpha$-$\hbox{Co}(\hbox{OH})_{2}$ (cubes) contains both octahedrally and tetrahedrally coordinated Co$^{2+}$ ions at the surface of the as-synthesized particles, while the $\beta$-$\hbox{Co}(\hbox{OH})_{2}$ (plates) contain only Co$^{3+}$ octahedrally coordinated at the surface of the particles (due to the oxyhydroxide surface). It is for these reasons that we end up with the surface terminations and shapes in $\hbox{Co}_{3}\hbox{O}_{4}$ i.e. the cubes have a higher Co$^{2+}$:Co$^{3+}$ ratio (1:2) at the surface with highly coordinated ions, and the plates have Co$^{3+}$ ions with a very low coordination and a small Co$^{2+}$:Co$^{3+}$ ratio (1:1).

### Electron microscopy

Scanning electron microscopy (SEM) and transmission electron microscopy (TEM) were performed at the Manitoba Institute for Materials. TEM was performed using a FEI TALOS TEM with an accelerating voltage of $200~\text{ keV }$. SEM was performed on a FEI Nova NanoSEM 450. Images were processed in Image J^43^.

### X-ray diffraction

X-ray diffraction (XRD) was performed with a BRUKER D8 Discover diffractometer. With a Cu $\hbox{K}_{\alpha }$ ($\lambda =1.5405$ Å) X-ray source at a voltage and current of $40~\text{ kV }$ and $40~\text{ mA }$, respectively. Patterns were collected from 10 to 90° with a resolution of 0.02° on a rotation stage with a knife edge and Ni $\hbox{K}_{\beta }$ filter. Visualization of the surface terminations by VESTA are performed using information obtained from the crystallography information files (CIF) from XRD refinements. We also utilize information from previous characterizations of Co3O4 surface planes characterized experimentally using high resolution TEM and theoretically using density functional theory (DFT) calculations^1,9–12^.

### Magnetometry

Magnetic measurements such as DC susceptibilities and magnetization vs field (*M* vs *H*) loops were performed using a Quantum Design MPMS-XL SQUID magnetometer. Samples were mounted in supracil quartz tubes to minimize background interference. DC susceptibility measurements were performed from 2 to 300 K in an applied field of $100~\text{ Oe }$. *M* vs *H* loops were performed at temperatures of 2, 5, 10, 15, 20, 25, 30, 50, 100, 200, and $300~\text{ K }$ with maximum applied fields of $\pm 50~\text{ kOe }$. Field-cooled hysteresis loops were performed at the same temperatures, with a cooling field of ${+}50~\text{ kOe }$.

### X-ray absorption spectrocopy (XAS) and X-ray magnetic circular dichroism (XMCD)

X-ray absorption spectroscopy and X-ray magnetic circular dichroism is collected with total electron yield (TEY) and total fluorescence yield (TFY) at the Advanced Photon Source at Argonne National Laboratory, utilizing the beamline 4-ID-C. Samples were placed on carbon tape mounted on the cold finger of the $70~\text{ kOe }$ superconducting magnet. Co $\hbox{L}_{3,2}$ edge spectra were normalized to the peak at $779.5~\text{ eV }$ and O K-edge spectra normalized to the peak at $529.5~\text{ eV }$. XMCD spectra were taken under applied fields of $\pm 50~\text{ kOe }$, and from these an artifact-free (AF) signal is obtained. Artifact-free spectra are obtained by collecting spectra under both positive and negative fields, then subtracting to obtain the portion present under both fields. This eliminates saturation from the TEY detection technique.

## Supplementary information

Supplementary material 1
