# Supplementary material for: Competing ferro- and antiferromagnetic exchange drives shape-selective Co3O4 nanomagnetism
Source: Sci Rep. 2020 Dec 2;10:20990. doi: 10.1038/s41598-020-77650-6 (PMC7710736; doi:10.1038/s41598-020-77650-6)
Supplement: Supplementary file 1 — Supplementary material 1 [file 41598_2020_77650_MOESM1_ESM.pdf]

Supplementary Information:

Competing Ferro- and Antiferromagnetic Exchange Drives Shape-selective Co3O4 Nanomagnetism

Michael Shepit,[a,] Vinod K. Paidi,[a] Charles A. Roberts,[b] Johan van Lierop,[a,]**

[a] Department of Physics & Astronomy, University of Manitoba, Winnipeg, MB, R3T 2N2, Canada

[b] Toyota Research Institute – North America, 1555 Woodridge Ave., Ann Arbor, MI 48105, United States.

* E-mail: shepitm@myumanitoba.ca, johan.van.lierop@umanitoba.ca

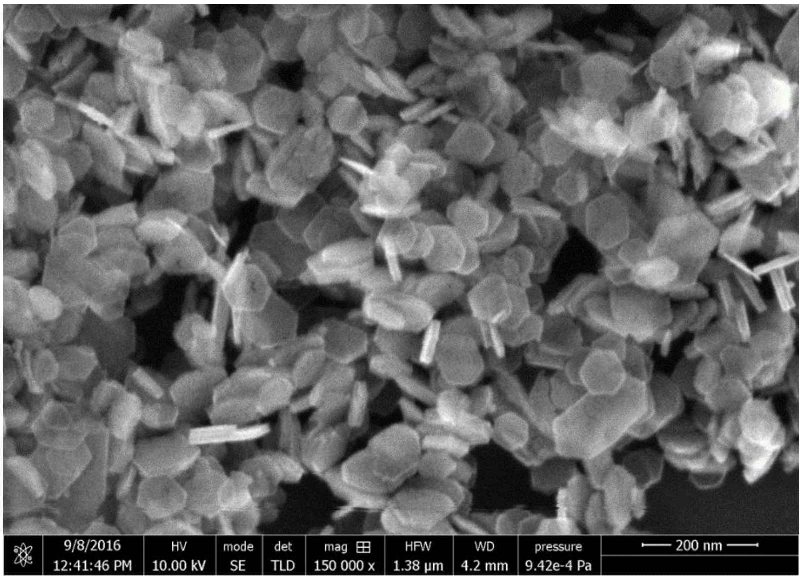

Figure S1: SEM Image of the hexagonal plates.

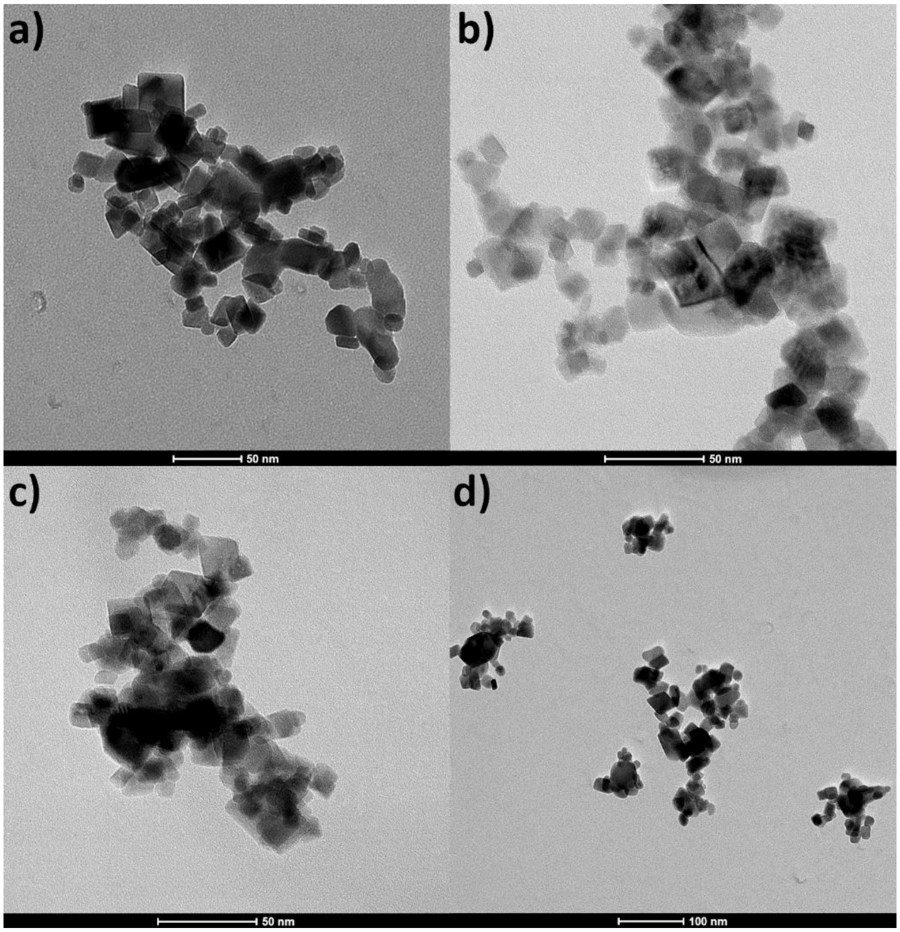

Figure S2: TEM Images of the cubes.

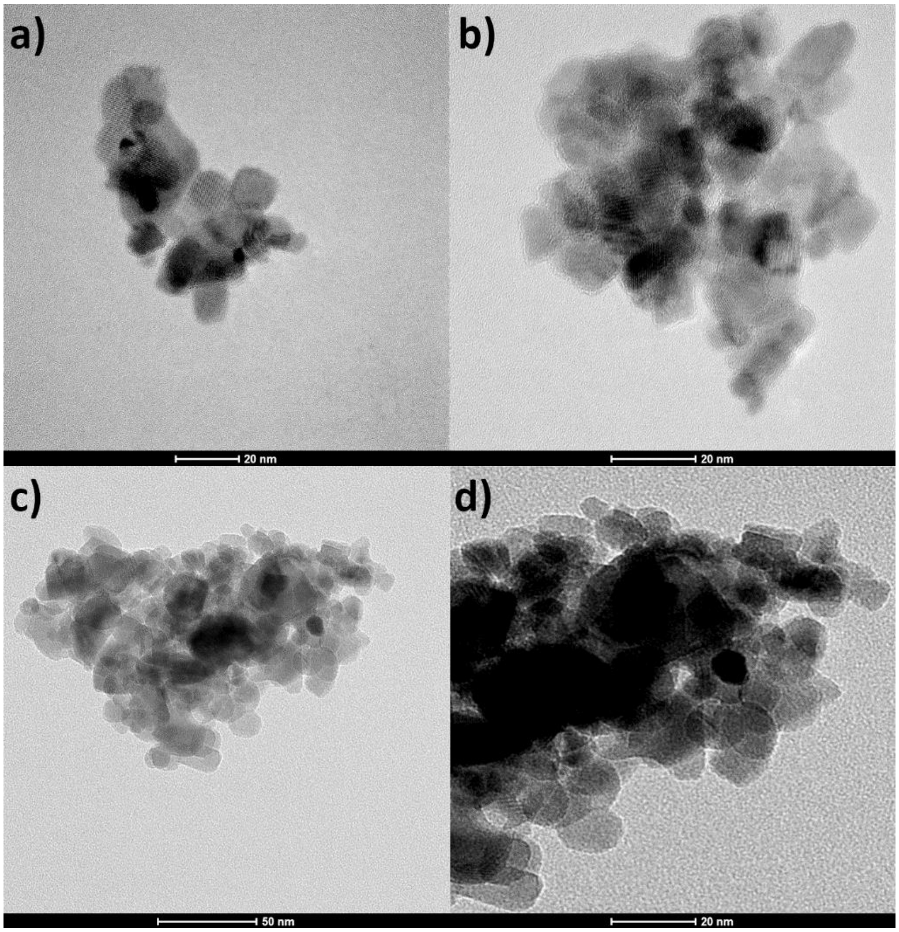

Figure S3: TEM Images of the spheres.

Values for the Co^{2+}:Co^{3+} ratio for the cubes and plates are obtained directly from the (100) plane and the (111) plane, respectively. Simulations of the each of the planes were done in VESTA. To calculate these parameters for the spheres, we used a weighted average of the three families of planes. For a given sphere there are 26 total planes, six from the {100}, 12 from the {110} and eight from the {111} families of planes. The Co^{2+}:Co^{3+} ratio can be found for each of the individual planes, allowing us to calculate a value for the spheres as:

$$[Co^{3+}]_{sphere} = \frac{6[Co^{3+}]_{100} + 12[Co^{3+}]_{110} + 8[Co^{3+}]_{111}}{26}$$

Where $[Co^{3+}]_{hkl}$ is the value of Co^{3+}/Co^{2+} ratio that is normalized to the relative unit cell areas for the different planes. Where the value of $[Co^{3+}]_{100} = [Co^{3+}]_{cube}$ is the same value used for the cubes, likewise for the plates.

The volume of a conventional unit cell in bulk Co_3O_4 is given by: $V_{bulk} = (0.8065)^3\ nm^3 = 0.53\ nm^3$. One unit cell contains 24 Co ions (16 Co^{3+} and 8 Co^{2+}) and the probe depth of the TEY measurements is 2-3 nm for the Co $L_{3,2}$ energies. Taking 2 nm leads to $\frac{2}{0.8065} 24 = 60\ Co\ atoms$ in the volume of probe depth across a surface area of one unit cell.

From VESTA we were able to obtain the surface unit cell areas, the number of Co ions at the surface in the unit cell, and the total number of ions for a probe depth of 2 nm for the same surface area (Table S1). The cubes expose the (100) plane, and thus there is six Co ions at the surface, with 60 ions in the probe depth of the measurement. Thus, $100 \times \frac{6}{60} = 10\%$ of the measurement is from surface Co. For the plates, the (111) plane is exposed, with $100 \times \frac{4}{50} = 7.9\%$.

Table S1: Surface parameters

Plane	Surface unit cell (nm^2)	Co^{2+} in surface unit cell	Co^{3+} in surface unit cell	Co ions in probe depth volume
**(100)**	0.65	2	4	60
**(110)**	0.46	2	2	42
**(111)**	0.57	2	2	50

Performing the same calculation for the (110) plane, and using a weighted average we determine the contribution at the surface to be 9.5% for the spheres. Thus the difference between the shapes is only 2% for TEY measurements, using the lower bound for the probe depth. A probe depth of 3 nm would decrease the overall surface contributions for each of the shapes.

TFY XAS and XMCD spectra for the different shapes are shown in figure S4 and S5. All shapes show very similar behaviour in TFY XAS but a vastly decreased amplitude in the TFY XMCD compared to TEY. This is expected for bulk sensitive measurements of an antiferromagnet such as Co_3O_4. The (TEY and TFY) XMCD spectra are each normalized to their respective XAS spectra. Thus, if the TEY XMCD measurements originated from the core of the particles we would expect to see a much larger signal in the corresponding TFY XMCD. The spectra are plotted on roughly the same scale (as TEY) for the y-axis to emphasize the lack of signal.

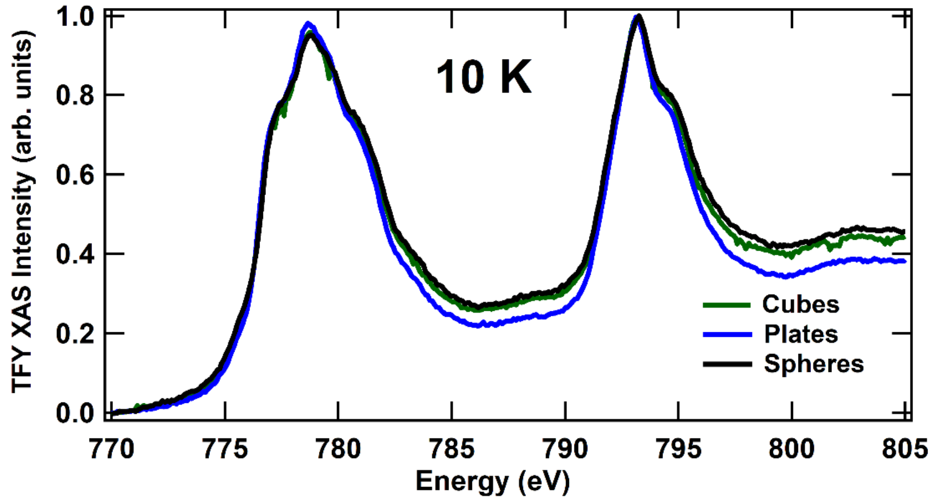

Figure S4: Normalized XAS for all the shapes at 10K using total fluorescence yield (TFY) detection. All Three shapes are Identical.

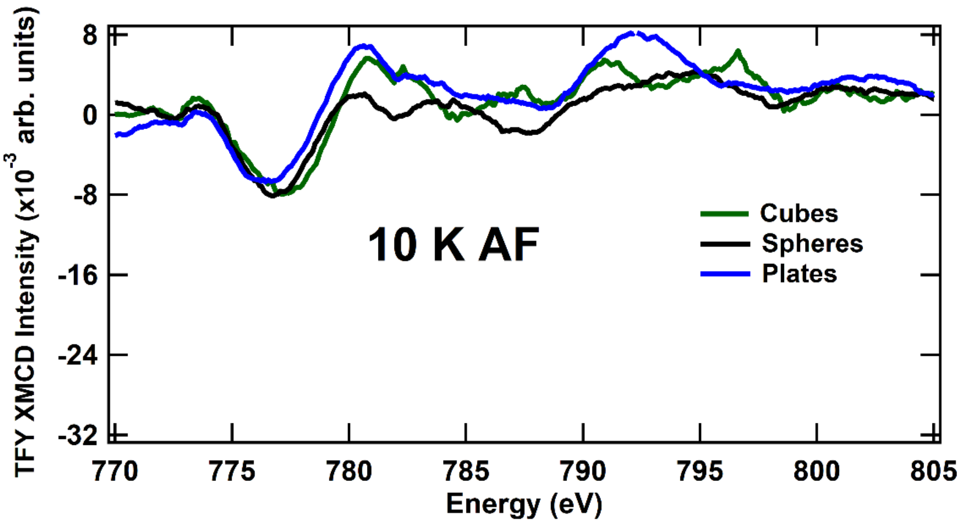

Figure S5: XAS normalized XMCD obtained under applied fields of $\pm$5 T using TFY detection shown for all shapes at 10K.

Figure S6 shows M vs H (quarter) loops for the cubes and plates at 10K. All of nanoshapes display a very clear paramagnetic component to the magnetization from the cores, while an additional paramagnetic component is present in the cubes and spheres above 10 kOe. This is further evidence of the spin-flop reconfiguration of the spins at the surface of those particles. The high-field linear component is a susceptibility, known as the high-field susceptibility χ_{HF}.

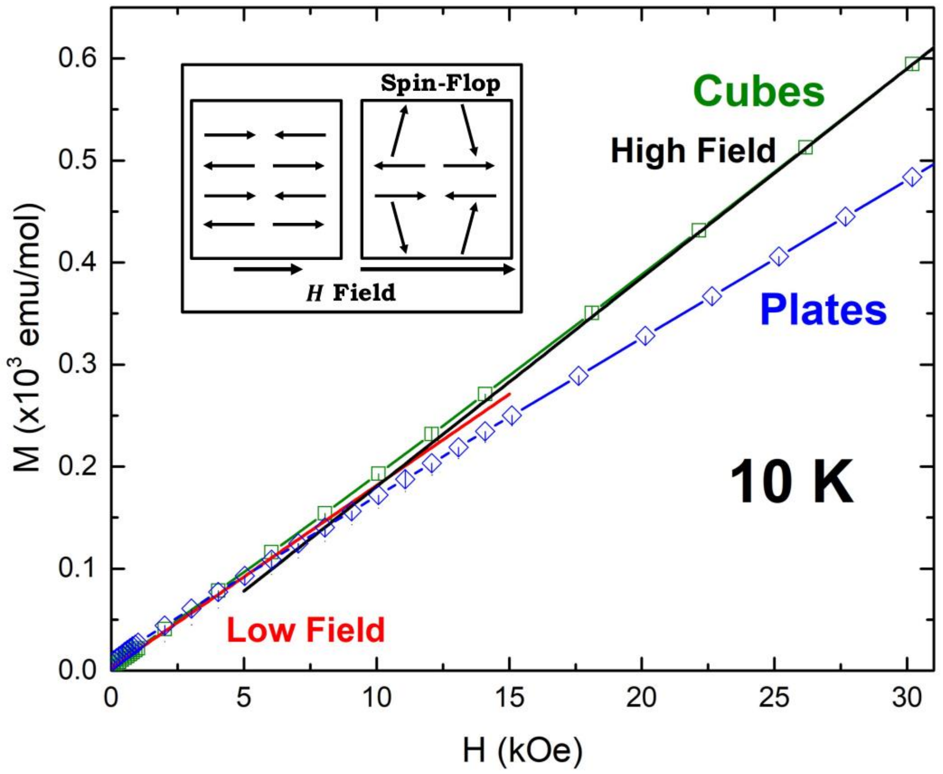

Figure S6: Quarter loops for the cubes and plates at 10K. Where the cubes show two linear components, a low-field, and high-field component.
